# Women and girls in resource poor countries experience much greater exposure to household air pollutants than men: Results from Uganda and Ethiopia

**DOI:** 10.1016/j.envint.2018.07.002

**Published:** 2018-10

**Authors:** Gabriel Okello, Graham Devereux, Sean Semple

**Affiliations:** a Respiratory Group, Division of Applied Health Sciences, University of Aberdeen, Aberdeen AB25 2ZP, UK; b Liverpool School of Tropical Medicine, Department of Clinical Sciences, Pembroke Place, Liverpool L3 5QA, UK; c Institute for Social Marketing, Faculty of Health Sciences and Sport, University of Stirling, Stirling FK9 4LA, UK

**Keywords:** Exposure assessment, Household air pollution, Biomass fuel smoke, Public health, Sub-Saharan Africa, Gender

## Abstract

Household Air Pollution (HAP) from burning biomass fuels is a major cause of mortality and morbidity in low-income settings worldwide. Little is known about the differences in objective personal HAP exposure by age and gender.

We measured personal exposure to HAP across six groups defined by age and gender (young children, young males, young females, adult males, adult females, and elderly) in rural households in two sub-Saharan African countries.

Data on 24-hour personal exposure to HAP were collected from 215 participants from 85 households in Uganda and Ethiopia. HAP exposure was assessed by measuring carbon monoxide (CO) and/or fine particulate matter (PM_2.5_) concentrations using five types of devices.

24 h PM_2.5_ personal exposure was highest among adult females with Geometric Mean (GM) and Geometric Standard Deviation (GSD) concentrations of 205 μg/m^3^ (1.67) in Ethiopia; 177 μg/m^3^ (1.61 GSD) in Uganda. The lowest PM_2.5_ exposures were recorded among young males GM (GSD) 30.2 μg/m^3^ (1.89) in Ethiopia; 26.3 μg/m^3^ (1.48) in Uganda. Young females had exposures about two-thirds of the adult female group. Adult males, young children and the elderly experienced lower exposures reflecting their limited involvement in cooking. There was a similar pattern of exposure by age and gender in both countries and when assessed by CO measurement.

There are substantial differences in exposure to HAP depending on age and gender in sub-Saharan Africa rural households reflecting differences in household cooking activity and time spent indoors. Future work should consider these differences when implementing exposure reduction interventions. There was a strong agreement between optical and gravimetric devices measurements although optical devices tended to overestimate exposure. There is need to calibrate optical devices against a gravimetric standard prior to quantifying exposure.

## Highlights

• First study in sub-Saharan Africa to examine differences in exposure to Household Air Pollution by age and gender
• 24h personal exposure measurement for PM_2.5_ and CO conducted in rural sub-Saharan Africa settings
• Comparison of measurements from optical and gravimetric devices
• Women and girls experience much higher household air pollutant exposures than men and boys

## 1 Introduction

The World Health Organisation (WHO) estimates that over 4 million deaths annually are attributable to exposure to Household Air Pollution (HAP) from biomass fuel smoke making it a leading cause of global mortality (Lim et al., 2012; WHO, 2016). Exposure to HAP is also a leading cause of disability, being associated with a range of illnesses including acute and chronic respiratory diseases, cardiovascular diseases, low-birth weight and cataracts (Gordon et al., 2014). HAP is generated from the incomplete combustion of biomass fuels such as wood, charcoal and crop residues and contains fine particulates often measured as Particulate Matter <2.5 μm in diameter (PM_2.5_) and gases such as Carbon Monoxide (CO).

Current estimates suggest that almost half of the world's population, including 700 million people in sub-Saharan Africa (SSA) rely on biomass fuels for cooking and heating, with burning typically taking place in simple three-stone traditional stoves or other similar inefficient arrangements (Mortimer et al., 2017). The global health burden resulting from exposure to the resulting HAP has placed interventions to reduce exposure to HAP high on the agenda of public health organizations and international development bodies (Bruce et al., 1998; Ezzati and Kammen, 2001; Gordon et al., 2014).

Currently there are a lack of high quality data on personal exposure to HAP in SSA with the limited data available generally collected by non-comparable, study-specific methods. Most epidemiologic studies have utilized indirect methods of exposure assessment such as comparing household fuel use or housing type as proxies for personal exposure (Bruce et al., 1998) whilst others have conducted fixed monitoring within homes to estimate personal exposures to HAP constituents (Fullerton et al., 2011; Keil et al., 2010; Klasen et al., 2015; McCord et al., 2017). Only a few studies have measured personal exposure to HAP in SSA (Ezzati et al., 2000; Van Vliet et al., 2013).

Research on exposure to HAP in Low and Medium Income Countries (LMICs) has tended to use a range of methods developed from occupational health or environmental sciences, making it difficult to compare the data because of short sampling times, poor calibration and positioning of devices (Gordon et al., 2014). Personal monitoring provides the opportunity to understand an individual's exposure in a specified microenvironment that may differ substantially from traditional methods used to generate population level exposure estimates, using fixed-site monitoring (FSM) networks and location of residence (Steinle et al., 2013).

To successfully design interventions to reduce exposure to HAP, it is important to understand the factors that influence an individual's exposure. Investigating variations of individual exposure to pollutants of concern by gender, age, household characteristics and household roles may provide intervention science with effective tools for the design of measures to reduce exposure to HAP.

We are not aware of any studies that have investigated differences in HAP exposure by age and gender in SSA using 24-hour average exposure measurements. The aim of this study was to determine if there are age and gender differences in HAP exposure by gathering personal exposure data from study participants in two rural settings in Uganda and Ethiopia.

## 2 Materials and methods

### 2.1 Study location and overview

A cross-sectional study was conducted in Kikati, Uganda and Kumbursa in Ethiopia, both villages are typical of rural villages in these countries. Kikati village is situated approximately 45 km from Kampala along the Kampala-Jinja highway. The village has approximately 400 households with most using biomass fuel for cooking purposes. Kumbursa is situated in Ude Kebele, Ada'a District in East Shoa Zone, Oromia National Regional State, Ethiopia. The village is located about 55.5 km south-east of Addis Ababa along the Addis Ababa – Adama old highway. The village has in total about 258 households with most using dung cake and crop residues for cooking.

In Kikati, houses are typically of brick construction whilst homes in Kumbursa are mostly constructed from soil/mud and wood/timber. Cow dung was the primary source of fuel used for cooking in the Ethiopian setting whereas wood was the primary fuel in the Ugandan homes. In addition to cooking food, roasting coffee beans was also common in Ethiopia. The main food cooked in Kumbursa homes was Injera (a sour flatbread common in Ethiopia) using traditional open stoves. The main food cooked in Kikati was Matooke (a banana based meal) with beans and groundnut sauce.

The study was conducted between February and June 2016 in Uganda and between July and September 2016 in Ethiopia – the rainy season in both regions. Ethical approval for the study was gained from the University of Aberdeen, College of Life Sciences Ethical Review Board (Application No. CERB/2016/2/1264).

### 2.2 Recruitment of participants

We conducted a multi stage sampling process where households in Kikati and Kumbursa villages were invited for a meeting with the help of community leaders and research assistants through market day announcements, worship day announcements and door to door messages. A brief presentation explained the objectives and methodology of the study to all those who voluntarily attended the meeting. An interpreter was used in Ethiopia. We also conducted demonstrations using the various instruments with volunteers from the meetings. From those households expressing an interest in participating, a random sample of those using biomass fuel as the main fuel for cooking and/or heating was taken to identify those who would participate in the study. The make-up of the household was confirmed by a personal visit and where possible one person from each of the six age-gender groupings was then invited to take part in the study. Age-gender groups were as follows: young children below 5 years of age (YC); young males (YM) and young females (YF) between 6 and 17 years; adult males (AM) and adult females (AF) aged between 18 and 49 years; and elderly adults (EL) aged 50 years or over. All participants provided written informed consent and for children, parental/guardian consent and assent of the child was obtained.

Based primarily on resource availability, a pragmatic target of 17 participants were recruited from each gender-age grouping in Ethiopia and Uganda. Measurement of personal exposure to PM_2.5_ and/or CO took place in a particular household, if one or more people in that household consented.

### 2.3 Household information

An interviewer administered questionnaire was completed with the head of the household to record details of household and kitchen size, fuel type, stove type, availability of windows, roof type, hours of cooking and time spent at home. Information on cooking patterns was also obtained. The questionnaire additionally contained questions regarding other non-cooking sources of HAP including tobacco smoking and use of candles/hurricane lamps. After appropriate training adult participants completed a simple time-activity dairy (TAD) for the day when the measuring device was worn. All TADs were checked with the participant.

### 2.4 Measurement of PM_2.5_ and CO

Personal PM_2.5_ exposure data were obtained from measurements over a 24-hour period using one or more of five types of air monitoring devices. The days of monitoring were chosen to be typical of the daily lives of the participants. The monitoring devices included: TSI SidePak AM510 Personal Aerosol Monitor (TSI Inc., CA, USA), the Dylos DC1700 (Dylos Inc., CA USA), RTI Micro Personal Exposure Monitor (MicroPEM) (RTI, NC, USA) and the Berkerely Air Group Particle and Temperature Sensor (PATS+) (Berkerely Air Group, CA, USA). To obtain 24 h of measurement, both the Dylos and SidePak instruments were connected to an Astro Pro2 power bank. Real-time carbon monoxide levels were measured every minute using a CO data logger (LASCAR EL-USB-CO) with a measurement range of 0–1000 ppm and a resolution of 0.5 ppm.

The Dylos 1700 measured the number of particles at minute intervals for two particle size ranges: >0.5 μm and > 2.5 μm; with particles between 0.5 and 2.5 μm being calculated by subtraction. Particle counts are expressed as a concentration per 0.01 ft^3^ of sampled air. The Dylos particle count for particle sizes ≤2.5 μm was calculated subtracting the >2.5 mm fraction from the total count number for particles >0.5 mm. Dylos particle count concentrations were converted to PM_2.5_ mass concentrations using a previously published conversion equation for combustion aerosol (Semple et al., 2015).

Two versions of Dylos devices were used for the study. One model was modified to have a slower fan speed and could measure to approximately 6000 μg/m^3^ whilst the standard version had a fan speed that provides maximum particle concentration data equivalent to about 1000 μg/m^3^.

The SidePak Personal Aerosol Monitor (AM510) measured airborne particle mass-concentration in mg/m^3^. TSI Sidepak AM510 was fitted was with a PM_2.5_ size selective impactor which removed particles larger than 2.5 nm. The device draws air through a size-selective PM_2.5_ impactor and uses a laser light scattering technique to quantify and log airborne concentrations of particulate matter every minute expressing the output in mg/m^3^ with a lower limit of detection of 0.001 mg/m^3^. At the start of each sampling session the Sidepak was zero calibrated using the manufacturer supplied High-Efficiency Particulate Air (HEPA) filter. The flow rate was set to 1.7 l/min using the TSI flowmeter. A calibration factor of 0.30 was adopted based on Jiang et al. (2011), however it should be noted that no single calibration factor applied before measurement would have been perfect as participants accessed different microenvironments which had different aerosols during the 24 h of measurement.

The PATS+ was deployed to measure real-time particulate matter (PM_2.5_) concentrations. The device had lower particulate matter detection limit 10 μg/m^3^ under most conditions and an upper particulate matter detection limit of 30,000 to 50,000 μg/m^3^. The device logged PM concentration, temperature, humidity, movement, and battery voltage. We used a logging interval of 1 min. The device was zeroed before and each sample measurement.

The MicroPEM device provides both time-resolved PM_2.5_ data via a micro-nephlometer and gravimetric PM_2.5_ concentration for the whole sampling period through filter samples collected on 3 μm PTFE 25 mm Teflon filters (Zefon International - Ocala, FL USA). The device operated at a flow rate of 0.44 l/min. Pre-and post-sampling filter weights, and field and lab blank weights, were determined in the temperature controlled Exposure Laboratory at the University of Aberdeen, UK. All filters were weighed twice, in both pre- and post-sampling on a ScalTec SBC 21 microbalance (Scaltec Instruments GmbH - Göttingen) maintained at the exposure laboratory, to acclimatize to the temperature and humidity of the lab for at least 24 h. Filters were weighed in the same lab under a stable temperature (22 °C).

The MicroPEM instruments were deployed together with other devices to measure PM_2.5_ for 24 h. After being retrieved, the real-time data were assessed with assistance from RTI and filters were posted back to the exposure laboratory for gravimetric measurement.

We experienced saturation with the Sidepak and the Dylos. Whenever an out of range reading was obtained, the highest concentration limit was used for the particular logged minute, i.e. 6000 μg/m^3^ for Sidepak, 1000 and 6000 μg/m^3^ for the lower concentration limit and the higher concentration limit Dylos devices respectively. The 24-hour concentrations measured by the optical devices were compared to the paired 24-hour measurements from the gravimetric devices.

As part of an additional study to compare data and to assess field-use of different devices, participants generally wore paired devices. In instances where a participant had two PM_2.5_ devices for 24 h, the gravimetric data are reported where available, followed by data from the Sidepak then Dylos and finally PATS+ in that order. This pragmatic hierarchy was employed to reflect the likely quality of PM_2.5_ data from gold-standard (gravimetric) through to lower-cost instrumentation. The time-resolved data from the micro-nephlometer of the MicroPEM device were not used due to download and interpretation problems.

Participants aged 18 years and above wore one or a pair of the particulate matter and carbon monoxide monitors around the waist or chest with the help of a strap or bag all of the day except during bathing and sleeping where devices were placed 1 to 2 m away from the participant. Participants aged between 15 and 17 years wore the devices in a similar manner to adults. Those aged between 2 and 15 did not wear the devices directly but instead kept moving the devices to the microenvironment they were in, either by themselves or with help of parents or older siblings. The devices were generally located within 1–2 m of the participant in each microenvironment. This group also received help from parents or older siblings when completing the TADs. For participants under 2 years, their exposure was assumed to be that recorded by a personal monitor worn by their mother or guardian.

### 2.5 Statistical analysis

Data were analysed using IBM SPSS version 24. The 24 h average PM_2.5_ and CO concentration data were generated for each participant together with minimum, maximum and the percentage of time over the WHO health-based guidance value of 25 μg/m^3^ and the US Environmental Protection Agency ‘Hazardous’ Air Quality guideline of 250 μg/m^3^. Minimum percentage of data required to compute representative 24 h averages was 96% (23 h). The Geometric Mean (GM) and Geometric Standard Deviation (GSD) for each age-gender group was calculated. Where the 24 h average carbon monoxide concentration of a participant indicated a value less than the limit of detection (LOD) of the carbon monoxide monitoring device, a value of one-half of the LOD (i.e. 0.25 ppm) was substituted for that participant in order to calculate the GM and GSD.

Two-way ANOVA was used to model HAP data and establish differences between gender-age groups in the two countries. Bonferroni and Tukey Post Hoc tests were used to establish the trend of differences between the gender-age groups across Ethiopia and Uganda. Optical and gravimetric paired devices were compared by using the average 24 h PM_2.5_ measurement. Agreement between the fine particulate matter measurement from the optical and gravimetric devices was achieved by use of the Bland-Atman plots/analysis. The spearman's rank correlation coefficient was used to quantify the relationship carbon monoxide and fine particulate matter.

## 3 Results

### 3.1 Participants in each age group and household characteristics

Of the 228 individuals who participated in the study, 215 provided valid air quality data relating to PM_2.5_ and/or CO. Eight participants had data for only 5 to 6 h due to accidental disconnection of the power cable. Data from a further five adults were excluded as they had not worn the device as requested. Detailed comparison of households' characteristics and the number of participants measured from each age group from each country are shown in Table 1. The mean number of individuals living in each household in Uganda was 6.0 (range 2–11) and 6.7 (range 3–11) in Ethiopia. Mean house volumes were 64.2 m^3^ (range 33.5 to 86.9 m^3^) in Ethiopia and 55.0 m^3^ (range 25.9 to 121m^3^) in Uganda. All the households in Ethiopia cooked indoors as opposed to Uganda where about one-fifth of the households cooked outdoors. The time activity diaries demonstrated that children aged <2 years in Ethiopia and Uganda spent 90% (21.6) hours and 86% (20.7 h) respectively in same microenvironment with their mothers.

**Table 1:** Number of participants measured from each age group and household characteristic comparison.

	Ethiopia	Uganda	Overall
Total number of households	258	400	656
Households	*n* = 41	*n* = 44	*n* = 85
Participants	n (%)		
Average no. per household	6	6.7	
No. of participants	*n* = 113	*n* = 102	*n* = 215
Young children (1–5 yrs)	19^a^ (17)	17^a^ (17)	36(17)
Young males (6–17)	19 (17)	16 (16)	35(16)
Young females (6–17)	19 (17)	17 (17)	36(17)
Adult males (18–49 yrs)	20 (18)	18 (18)	38(18)
Adult females (18–49 yrs)	17 (15)	18 (18)	35(16)
Elderly (+50 yrs)	19 (17)	16 (16)	35(16)
Type of windows			
No window	2 (5)	7 (16)	9 (11)
Wood	19 (46)	28 (64)	47 (55)
Glass	14 (34)	8 (18)	22 (26)
Metal	6 (15)	1 (2)	7 (8)
Kitchen characteristics			
Kitchen			
Present	41 (100)	36 (82)	77 (91)
Absent	0 (0)	8 (18)	8 (9)
Type of windows			
None	36 (88)	14 (33)	53 (62)
Wood	5 (12)	4 (8)	9 (11)
Glass	0 (0)	12 (28)	11 (13)
Metal/iron sheet	0(0)	14 (31)	12 (14)
Biomass fuel assessment			
Cooking location			
Indoors	40 (98)	36 (82)	76 (89)
Outdoors	1 (2)	8 (18)	9 (11)
Primary cooking fuel			
Firewood	10 (25)	40 (91)	59 (69)
Dung	29 (70)	0 (0)	20 (24)
Crop residues	2 (5)	4 (9.0)	6 (7)

a Infants (<2 yrs) were 4 in Ethiopia and 3 in Uganda. Their exposure was assumed to be that worn by the mother or guardian.

### 3.2 Agreement between gravimetric and optical devices

**Fig. 1:**
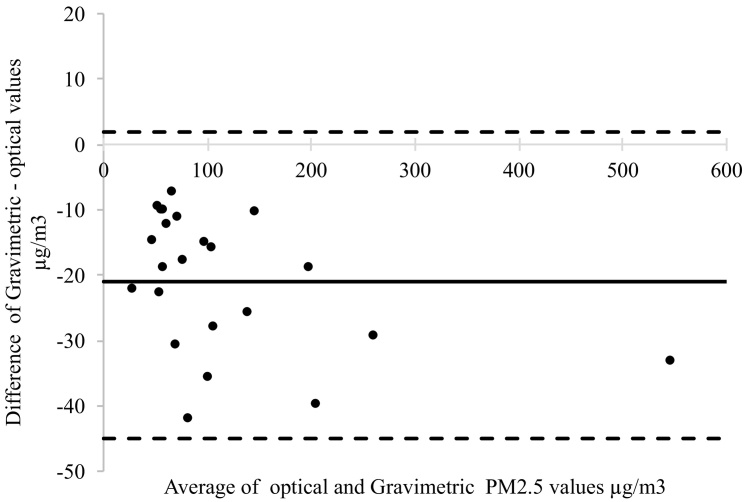
Bland-Altman plot demonstrating agreement between optical and gravimetric PM_2.5_ values.

As part of the study to compare data from the various devices, participants generally wore paired devices. Data to compare the PM_2.5_ exposures from the different device was obtained from measurements over a 24-hour period using paired PM air monitoring devices. Table 2 shows the number of pairs in each of the countries. Bland–Altman plots were used to compare the gravimetric measurements of PM2.5 (from the filters used in the MicroPEM devices) with the measurements from optical device (Fig. 1) and agreement between measurements from the optical devices (Supplementary data).

**Table 2:** Showing pairing of the fine particulate matter measuring devices in Uganda and Ethiopia.

Type of pairing	No. of 24 h pairings	
	Uganda	Ethiopia
SidePak - Dylos	18	10
SidePak - Lascar	30	20
Dylos - Lascar	31	37
Dylos - MicroPEM	9	3
SidePak - MicroPEM	7	1
Lascar - MicroPEM	7	5
PATS+ - Lascar	0	21
SidePak - PATS+	0	8
Dylos - PATS+	0	6
PATS+ - MicroPEM	0	2

The mean difference for 12 paired sets of measurements using the MicroPEM and Dylos was 14.7 μg/m^3^. For 8 sets of paired sets of measurements using the MicroPEM and Sidepak, the mean difference was 27.9 μg/m^3^. For 2 sets of paired sets of measurements using the MicroPEM and the PATS+, the mean difference was 38.9 μg/m^3^. These data suggested that the optical devices tended to overestimate exposure when compared with the gravimetric methods and this was confirmed by Bland Altman analysis (Fig. 1) that indicated that the mean difference between the optical and gravimetric devices was −21 μg/m^3^ with limits of agreement of −45.0 to 1.9 μg/m^3^. No data points were outside the 95% limits of agreement. Bland-Altman plots used to compare agreement between collocated optical devices indicated acceptable agreement between measurements with Sidepak and Dylos showing 96.3% (*n* = 186), Sidepak and PATS+ showing 97.4% (*n* = 190); and Dylos and PATS+ showing a 99% (*n* = 117) of the values within the 95% acceptable range (See supplementary documents for the plots).

Our results from device comparison show that optical devices namely Sidepak, Dylos and PATS+ are likely to overestimate exposure compared to the gravimetric devices.

### 3.3 Correlation between carbon monoxide and fine particulate matter

We obtained 151 simultaneous 24-hour measures of CO and PM_2.5_ in Uganda (68) and Ethiopia (83). There was strong correlation between CO and PM_2.5_ measures for Lascar-MicroPEM paired devices (Spearman rank correlation coefficient ρ = 0.74, ρ = 0.004), Lascar-Sidepak paired devices (ρ = 0.80, *p* < 0.001) and moderate correlation for Lascar-Dylos (ρ = 0.61, ρ < 0.001), and Lascar-PATS+ (ρ = 0.50, ρ < 0.001) paired devices.

### 3.4 Comparison of mean 24 hour PM_2.5_ and CO in Ethiopia and Uganda

A two-way ANOVA between Ethiopia and Uganda showed that exposure to PM_2.5_ was significantly higher in Ethiopia (*p* = 0.041). As outlined in Table 3, overall PM_2.5_ exposure concentrations were highest among the adult females with GM concentrations (GSD) 177 μg/m^3^ (1.61) and 205 μg/m^3^ (1.67) in Uganda and Ethiopia respectively. Young females experienced the second highest concentrations with GM concentrations (GSD) of 118 (1.44) and 134 μg/m^3^ (1.94) in Uganda and Ethiopia respectively. The lowest exposures measured occurred among young males with GM (GSD) values of 26.3 μg/m^3^ (1.48) and 30.3 μg/m^3^ (1.89) in Uganda and Ethiopia respectively. There was a significant difference in 24 h PM_2.5_ exposure between young males and young females (*p* < 0.001) in Uganda; and similarly, between young males and young females (*p* < 0.001) in Ethiopia.

**Table 3:** Comparison of mean 24 hour PM_2.5_ in μg/m^3^ and CO in ppm concentrations among the six age groups in Ethiopia and Uganda.

Group	Uganda		Ethiopia	
	PM_2.5_ GM (GSD) μg/m^3^	CO GM (GSD) ppm	PM_2.5_ GM(GSD) μg/m^3^	CO GM (GSD) ppm
Infants	80.2 (1.34); *n* = 17	0.64^d^ (2.12); *n* = 13	97.0 (1.89); *n* = 17	2.4^d^ (3.07); *n* = 11
Young males	26.3^a^ (1.48); *n* = 16	0.02^c^ (3.67); *n* = 12	30.3^a^ (1.89); *n* = 20	0.48^c^ (3.07); *n* = 18
Young females	117.6^a^(1.49); *n* = 17	0.81^c^, ^d^ (3.83); *n* = 9	134.4^a^ (2.16); *n* = 18	2.52^c^, ^d^ (2.28); *n* = 13
Adult males	32.3^b^ (1.97); *n* = 17	0.17^c^ (4.34); *n* = 12	40.5^b^ (2.55); *n* = 18	0.34^c^ (3.27); *n* = 14
Adult females	177.2^b^ (1.61); *n* = 19	0.95^c^, ^d^ (3.26); *n* = 19	205.4^b^ (1.67); *n* = 20	3.98^c^, ^d^ (1.70); *n* = 17
Elderly adults	63.9 (2.03); *n* = 16	0.54 (3.07); *n* = 10	45.6 (1.79); *n* = 19	0.26 (2.95); *n* = 19

a Shows significant difference in PM_2.5_ exposure (*p* < 0.01) between young males and young females in both countries.

b Shows there is significant difference in PM_2.5_ exposure (*p* < 0.001) between adult males and adult females in Uganda and Ethiopia.

c Show significant difference in CO exposure (*p* < 0.001) between young males and females; and adult males and adult females in both countries.

d Shows significant difference in CO exposure (*p* < 0.001) between similar gender age groups across both countries.

There was also a significant difference in 24 h PM_2.5_ exposure between adult males and adult females (*p* < 0.001) in Uganda; and similarly, in Ethiopia, between adult males and adult females (p < 0.001).

There was no significant difference in 24 h PM_2.5_ exposure between children and adults (*p* > 0.001) in Ethiopia; whereas Uganda results showed a significant difference between children and adults (*p* < 0.001).

There was significant difference in CO exposure between similar gender-age groups namely young children, young females and adult females with *p* values of 0.008, <0.001 and 0.006 respectively. Similar exposure profiles and age-gender differences were evident from the carbon monoxide results: measurements were highest among adult females and lowest among young males. The difference in GM CO concentrations between adult females and adult males was approximately 5-fold in Uganda and 11 times in Ethiopia.

The two-way ANOVA showed no significant differences in 24 h CO exposure between Young children and young females, young children and adult females, young males and adult males, young males and elderly, and young females and adult females across the two countries. Bonferroni and Tukey Post Hoc tests showed similar trend of differences between the gender-age groups across Ethiopia and Uganda.

#### 3.4.1 Comparison of 24 h mean PM_2.5_ concentrations among the various gender/age groups in Ethiopia and Uganda

**Fig. 2:**
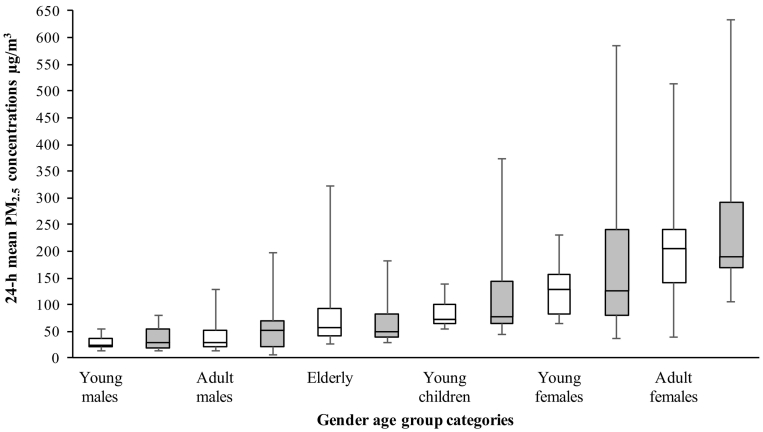
Comparison of 24 h mean PM_2.5_ concentrations among various age groups in Ethiopia and Uganda.

Fig. 2 shows the comparison between the 24 h PM_2.5_ concentrations among the various gender/age groups in Ethiopia and Uganda respectively. The PM_2.5_ concentrations in each age group are also compared with the WHO 24 h PM_2.5_ guidance limit of 25 μg/m^3^ (WHO, 2016). Average 24 h PM_2.5_ concentrations were generally higher in Ethiopia for similar gender age groups compared to Uganda. Results also show that overall average exposure by all gender age groups across the two countries was higher than the WHO 24 h guideline limit.

Box plot of average 24 h PM_2.5_ concentrations by gender-based age group in Uganda and Ethiopia. Dashed line shows the WHO 24 h PM_2.5_ guidance limit which is 25 μg/m^3^.

#### 3.4.2 Temporal data on exposure of adult females

**Fig. 3:**
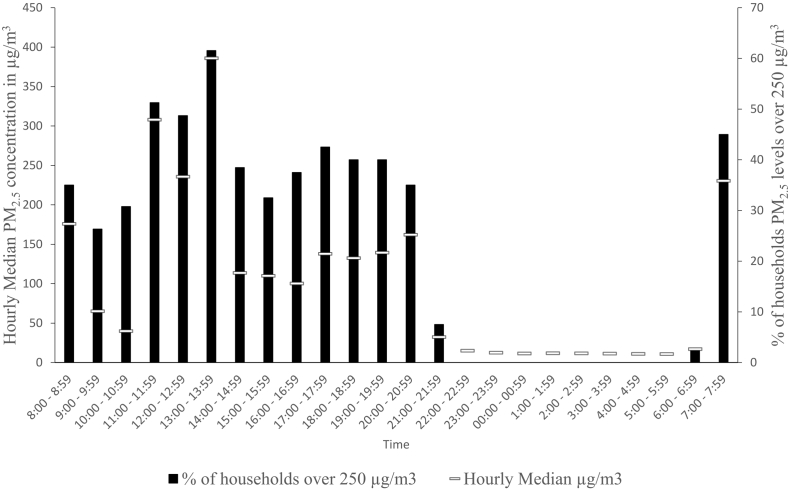
Median PM_2.5_ concentration levels among adult females throughout the day and percentage of homes with PM_2.5_ levels exceeding 250 μg/m^3^ in each hour in Ethiopia and Uganda (*n* = 35).

Fig. 3 illustrates the hourly median PM_2.5_ concentrations aggregated from 19 and 17 adult females in Ethiopia and Uganda. The graph also displays the percentage of participants in each hour time period that were exposed to PM_2.5_ concentrations that would be deemed very unhealthy (>250 μg/m^3^) under the US-EPA PM_2.5_ air quality index (AQI) breakpoint (US EPA, 2016) and prolonged exposure should be avoided.

Most adult females experienced high concentrations in the morning, afternoon and evening. The highest intensity exposure where PM_2.5_ concentration in homes was >250 μg/m^3^ among adult females in both countries were experienced from 1 to 1:59 pm (61.5%) followed by 11 to 11:59 am (51.3%) and then 12 noon to 12:59 pm (48.7%). 45% of the households experienced PM_2.5_ concentrations above 250 μg/m^3^ between 7 and 8 am.

The highest hourly median PM concentration 386 μg/m^3^ was recorded from 1 pm to 2 pm followed by 308 μg/m^3^ recorded between 11 am to 12noon. The lowest hourly median of PM_2.5_ concentration 11 μg/m^3^ was recorded between 4 am and 6 am.

**Fig. 4:**
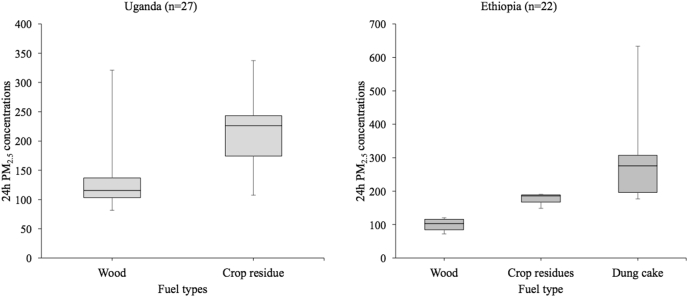
Comparison of 24 h mean PM_2.5_ exposure from primary cook in Ethiopia and Uganda according to fuel type.

An assessment on the effect of fuel type used for cooking in the household (Fig. 4) showed that women who cooked with dung cake had highest exposures with median PM_2.5_ exposures of 276.1 μg/m^3^ compared to 185.7 μg/m^3^ and 119.9 μg/m^3^ exposure experienced by those who cooked with crop residues and wood respectively in Ethiopia. Women who used crop residues also had higher exposures to fine particulate matter with median PM_2.5_ exposures of 226.3 μg/m^3^ compared to those using wood who had median exposures of 115.7 μg/m^3^ in Uganda. We only found one male as the primary cook in Ethiopia and two males in Uganda who at times reported helping with cooking. The males however did not participate in the cooking on the day of the 24-hour measurement.

## 4 Discussion

This is the first study to report data on personal exposure to HAP for rural households in Uganda and Ethiopia and the first to consider differences in exposure by age and gender for PM_2.5_ measurement in SSA. We found that women and girls had much higher exposures than men.

Our study is similar to the work Saksena et al. (1992) and Ezzati et al. (2000). These studies measured concentration of air pollutants in different microenvironments in the Himalayas and Kenya respectively, using direct monitoring. It should be noted that both measured different size fractions of particulate matter; Saksena measuring Total Suspended Particulate (TSP) and Ezzati measuring PM_10_. The current study provides detail on age and gender differences based on personal exposure monitoring for full 24-hour periods thus extending on the literature on HAP exposure assessment in low-income rural settings.

Previous work in SSA has mostly utilized fixed site monitoring air quality in urban areas (Dionisio et al., 2010; McCord et al., 2017; Zhou et al., 2011) and rural settings (Amegah and Agyei-mensah, 2017; Keil et al., 2010; Tumwesige et al., 2017), however only four studies have conducted personal monitoring (Ezzati et al., 2000; Titcombe and Simcik, 2011; Van Vliet et al., 2013; Yamamoto et al., 2014). These are summarized in Table 4.

**Table 4:** Selection of studies that have measured PM and/or CO in households burning biomass in sub-Sahara Africa.

Country	First author (year)	Setting	Sample size	Fuel	Monitoring	Exposure	Exposure concentrations
Burkina Faso	Yamamoto et al. (2014)	Urban		Wood and charcoal	Area Personal	24 h PM_10_ 24 h CO	Personal CO = 3.3 ppm, 95% CI 2.8–3.8 ppm, *n* = 145; Area CO = 16.9 ppm, 95% CI 12.3–21.4 ppm, *n* = 119
Ghana	Van Vliet et al. (2013)	Rural	421 households 36 sub sample	Wood, charcoal	Fixed site, Personal	24 h PM_2.5_ 24 h Black carbon	PM_2.5_ = 446.8 μg/m^3^, Black carbon = 14.5 μg/m3 Fixed site monitoring; PM_2.5_ = 128.5 μg/m^3^, Black carbon = 8.8 μg/m^3^ for personal monitoring
Tanzania	Titcombe and Simcik, 2011	Urban and rural	9 households (biomass)	Firewood charcoal	Personal	7 to 8 h PM_2.5_	Mean PM_2.5_ = 588 μg/m^3^ for charcoal and PM_2.5_ = 1547 μg/m^3^ (SD = 287 μg/m^3^) for open wood fires
Kenya	Ezzati et al. (2000)	Rural	55 households (345 individuals)	Wood Charcoal	Fixed site	14 h PM_10_	0–5 years mean PM_10_ = 1317, SD = 1188 (female) and mean PM_10_ = 1449, SD = 1067 (male); for 6–15 years mean PM_10_ = 2795, SD = 2069 (female) and Mean PM_10_ = 1128, SD = 638 (male); for 16–50 years Mean PM_10_ = 4898, SD = 3663 (female) and Mean PM_10_ = 1018, SD = 984 (male); for >50 years mean PM_10_ = 2639, SD = 2501 (female) and mean PM_10_ = 2169, SD = 977 (male).
Gambia	Dionisio et al. (2008)		13 households	Firewood charcoal	Fixed site	48 h PM_2.5_ 48 h CO	Mean PM_2.5_ = 361 μg/m^3^ (SD = 312 μg/m^3^), Mean CO = 4.69 mg m^−3^ (SD = 4.81 mg m^−3^) cooking area; Mean PM_2.5_ = 219 μg/m^3^ and CO = 1.5 ppm children, PM_2.5_ = 275 μg/m^3^ and CO 2.4 ppm mothers

Key: CO = carbon monoxide; PM_10_ = particulate matter; PM_10_ = particulate matter with aerodynamic diameter <10 μm; PM_2.5_ = particulate matter with aerodynamic diameter <2.5 μm; SD = standard deviation; CI = confidence interval.

Van Vliet et al. (2013) reported exposure results that are broadly comparable to our 24 h exposure average PM_2.5_ concentrations of 118 μg/m^3^ in Uganda, 134 μg/m^3^ in Ethiopia and for adult females 205 μg/m^3^ and 177 μg/m^3^ in our study in Ethiopia and Uganda respectively.

Young children had exposures linked to their mothers 24-h CO exposure data of 0.64 ppm and 1.62 ppm for children and mothers in Uganda; and 2.4 ppm and 3.98 ppm for children and mothers in Ethiopia. This is likely to be attributable to the time young children spent in the same microenvironments with their mothers. The closeness of young children to their mothers or care givers should always be considered during implementation of exposure reduction programs or policies. The overall exposure of young children will largely be determined by the mothers and/or care givers involvement in cooking and the time young children spend near them.

Our study demonstrates that adult females had considerably greater exposure compared to adult males of the same age group category (4.5 times greater in females for PM_2.5_ and 5.4 times greater for CO in Uganda; 4.4 (PM_2.5_) and 5.1 (CO) times in Ethiopia). These trends are similar to a study conducted by Ezzati et al. (2000) that reported young and adult females to have the highest exposure among the age groups and considerably greater exposures to HAP than males of the same age group (2.5 and 4.8 times, respectively) in Kenya.

Measuring PM_2.5_ and CO for 24 h enabled us to measure the concentration experienced by the various participants throughout the full day and makes our data easier to compare with future studies that also measure for 24 h periods. From our data, we found that PM_2.5_ concentrations can be higher even after 8.30 pm depending on the household/participant activity and the time a participant slept. For example, some coffee ceremonies in Ethiopia which involved roasting of coffee and burning of incense, took place after 7 pm in the house. Therefore, it is desirable to carry out at least 24 h personal exposure measurements.

Fixed site monitoring is also likely to lead to exposure misclassification as people move about from one micro-environment to another. This spatial variability makes it difficult to rely on time-activity diaries especially since people sometimes forget the exact times they were in specific microenvironments.

Titcombe and Simcik (2011) reported high personal exposure for the primary cooks using biomass fuel in a study carried out in Tanzania (Table 4). It is difficult to compare our results with these given that their study collected data for just 8 h and typically during high-exposure cooking periods. Again, this highlights the need for exposure measurement that spans a full 24-hour period to enable direct comparisons and to enable consideration of exposures within the context of 24-hour health based PM_2.5_ guidelines such as the WHO value of 25 μg/m^3^.

Yamamoto and colleagues utilized Gastec CO colour dosimeter tubes to estimate both area and personal concentrations and found much lower personal CO concentrations compared to area monitoring for 24 h measurements (Yamamoto et al., 2014). The average personal 24 h CO concentrations are in similar range to the concentrations in our study (2.4 ppm and 3.98 ppm for young females and adult females in Ethiopia).

Our study shows that adult females were the gender-age group exposed to the highest HAP concentrations in both Ugandan and Ethiopian rural households. The exposure of this group is, on average, six to seven times higher than the WHO 24-hour guidance of 25 μg/m^3^. These concentrations of PM_2.5_ are similar in scale to average PM_2.5_ levels (246 μg/m^3^) measured in bars where smoking was permitted in Scotland before comprehensive smoke-free legislation was enacted as reported by Semple et al. (2007).

Adult females in both countries had the leading role in domestic cooking usually being helped by the young females. This role subjected women to several periods of intense cooking smoke exposure each day thus leading to the high exposures. The adult males were engaged in mostly farming and small scale businesses in the trading centres and spent much of their time away from the home.

These results help increase understanding of the dynamic relationships between the different age groups in different time and locations. These data may be of use in helping researchers develop a better understanding of the timing and processes involved in individual exposure to HAP by taking account of all environments in which people spend time. For example, interventions to reduce emissions from stoves may impact much more on the exposure of females than males.

It is worth noting that average 24 h PM_2.5_ exposures were higher for each gender based age group in Ethiopia compared to Uganda. This could be attributed to difference in fuels used, poorer ventilation levels and a comparative lack of windows in the kitchen areas in Ethiopia (88% of Ethiopian homes had no windows in the kitchen area compared to 33% in Uganda). Higher PM_2.5_ and CO levels in Ethiopia across age groups could also be attributed to the “coffee ceremony” process where coffee beans are roasted over traditional stoves. This was often combined with burning incense in a small burner made from clay. The ceremony was generally carried out in most Ethiopian households in the morning and evenings.

Our study showed a 5-fold and 11-fold difference for 24 h CO exposure between adult males and females in Uganda and Ethiopia respectively yet PM_2.5_ the difference is approximately 5-fold in both countries. One reason could be attributed to the use of dung cake which was predominant source of cooking fuel in Ethiopia. Combustion of dung cake has been found to have high emission of CO ranging 14–29 g kg^−1^ compared to 11–12 kg^−1^ of wood (Venkataraman and Rao, 2001).

Chakraborty et al. also reported higher CO content produced from dung than wood in their work to assess effect of exposure to biomass smoke on various health status (Chakraborty et al., 2014). Another reason is, burning animal dung efficiently is more difficult than burning wood efficiently due to conversion efficiency. The frequent evening rituals/ceremony of making coffee could have also been a major factor. The coffee ritual involves roasting beans for some time which occurs more often (4 to 5 times a week).

A general comparison of fine particulate matter exposure between children (0 to 17 year) and adults (18 to 49 years) showed a significant difference in Uganda but not in Ethiopia. This further justifies the needs of gender-age quantification to avoid misclassification.

We were also able to conduct paired measurements in the field. Our results indicated that optical devices (Sidepak, Dylos and PATS+) generally overestimated exposure compared to the paired gravimetric device (MicroPEM). Although the optical devices tended to overestimate exposure by 21 μg/m^3^ on average and the data points were within the 95% limits of agreement, the overestimate was as high as ~40 μg/m^3^ which is important if a study only used optical devices to quantify exposures and represents one of the limitations of using optical instead of gravimetric devices. It is therefore important to always calibrate the optical measuring devices against a gravimetric device. It is worth noting that there was a strong agreement between optical and gravimetric fine particulate matter measurements. Researchers can therefore quantify exposure in rural SSA settings using optical devices thus avoiding many of the complexities surrounding gravimetric devices like access to an exposure laboratory, high sensitivity scale, pre and post weighing of filters etc.

There was a weak correlation between CO and PM_2.5_. We emphasize that this was observed for personal exposure among the different gender-age groups. This should not be generalized with correlation during cooking episodes.

The strengths of this study include the large dataset of over 5160 h of measurement across 215 subjects in two countries. This is one of the largest datasets of personal exposure to HAP in SSA. Previous studies that have measured personal exposure to HAP in SSA include Ezzati and Kammen (2001). Ezzati et al. measured 14 h personal exposure to PM_10_ and CO for 345 participants in 55 households in Kenya. Two hundred ten 14 h days gives an approximate total duration of 2940 h of measurement for the dataset. Van Vliet and group had a much smaller sample size (36 homes) for exposure measurement in rural Ghana (Van Vliet et al., 2013).

Another strength of the study was the use of personal monitoring which accounted for the individual exposure patterns in different microenvironments as opposed to fixed site monitoring which is likely to give higher concentrations as reported by Van Vliet et al. (2013).

24-hour measurement is a particular strength of the work as it presented a complete day in the life of each participant in a specific household. Although this represents a considerable improvement over shorter sampling periods it is still possible that wearing devices led to behaviour modification for some participants in the study. Some participants may have spent more time discussing the devices with friends, work colleagues and school friends and so their daily pattern of activities may have altered. Future work should consider gathering data over longer periods, use of ultrasound personal locators UC Berkeley Time Activity Monitoring System UCB-TAMS and Global Positioning Systems (GPS) to see if personal exposures change once the ‘novelty’ of wearing such devices wears off. However, there are considerable practical problems particularly around power supply, associated with measuring exposures for extended time periods.

Our age cut-offs were arbitrary and were intended to reflect differences in activities and behaviours but we acknowledge that our definition of young child (0–5 years) masks large differences between infants (<2 y) who spend almost all their time close to their mother, and children aged 4–5 who may have much greater freedom to move in and out of the home setting. Similarly, our decision to include both male and female participants in a single elderly age group may have hidden important differences here with elderly women perhaps more likely to provide some assistance and experience during cooking activity. Elderly men are more likely to be involved in work around the homestead like sweeping and weeding around the compound.

The study was restricted to rural households. Research has shown that many urban households in developing countries also use biomass fuels for cooking with the proportions of use generally varying according to socioeconomic status. Zhou et al. (2011) reported high PM concentrations in low socioeconomic status slums in Ghana. It is possible that urban households experience even higher PM_2.5_ exposures due to the additional outdoor air pollution from vehicle and industrial emissions in these settings. Future work should gather information on the differences in exposure profiles by age/gender in urban homes in SSA.

Although Kikati and Kumbursa are similar to many rural settings characterised by lack of access to electricity, heavily relying on biomass for cooking and heating, we acknowledge they may not represent all the villages in sub-Saharan Africa. However, our data is consistent with social science and other studies.

## 5 Conclusions

Personal exposures to HAP are high in rural homes in Ethiopia and Uganda. Women and girls bear the brunt of these high exposures, an issue which is frequently addressed in the social sciences literature but not often quantified from an environmental and health perspective. Overall, we found an approximate five-fold difference in the PM_2.5_ exposure of adult males and adult females reflecting household cooking activity and time spent indoors.

There are substantial differences in personal exposure to HAP from biomass fuel smoke depending on the age and gender of an individual in sub-Saharan Africa rural households. Exposure to HAP in a single household can vary from day to day because of fuel characteristics such as moisture content or density, air flow, cooking method, fuel stacking is being practiced. Individual exposure can also vary because of the different activities carried out on different days including church days, market days, going to school, etc.

It is vital for future work to consider these differences in exposure to HAP across the life-course and to characterise age and gender differences when implementing exposure reductions interventions. Health education interventions should target females and explain the benefits to their health of reducing HAP concentrations.

There was an agreement between measurements from optical and gravimetric devices though optical devices overestimated exposure. There is need to calibrate an optical device against a gravimetric device prior to quantifying exposure.

## Funding

This work was funded by the Medical Research Council, UK (Grant no. MR/L009242/1) and is part of a larger project (BREATHE - Biomass Reduction and Environmental Air Towards Health Effects in Africa) focusing on quantifying and reducing the health effects of household air pollution.

## Appendix A Supplementary data

Supplementary material Image 1
